# The digital transformation of healthcare: a national cross-sectional study on usage, patterns, attitudes, and barriers across social groups in Israel

**DOI:** 10.3389/fpubh.2025.1735502

**Published:** 2026-01-12

**Authors:** Orly Toren, Galit Madar, Sima Reicher

**Affiliations:** 1 Department of Nursing, Ono Academic College, Kiryat Ono, Israel; 2 School of Communication, Ariel University, Ariel, Israel

**Keywords:** attitudes, digital usage barriers, health literacy, high-level technology, low-level technology, online healthcare services

## Abstract

**Background:**

Online health services (OHS) have emerged as a response to healthcare challenges, offering a way to enhance system efficiency. Despite their numerous advantages, studies reveal varying efficacies among populations with differing sociodemographic characteristics. In this study, we describe OHS usage and its characteristics, examine knowledge, attitudes, barriers, and usage patterns in different groups in Israel, and present a predictive model for OHS consumption.

**Methods:**

Using a random representative sample stratified by sex, ethnicity, age, and religious affiliation, a cross-sectional study was conducted in Jewish and Arab populations in Israel. Sampling was further stratified by ethnicity and geographical region. Data were collected via an online questionnaire administered through iPanel records.

**Results:**

The sample comprised 2001 participants with an average age of 47 years; about half were women. The research differentiated between low- and high-level technologies. Participants reported an absence of technological or emotional barriers and were more familiar with low-level technology and used it more frequently. The perceived efficacy of OHS was high, but some participants preferred face-to-face treatment. Predictors of familiarity with and use of high-level OHS included being male, Arab, insured by the Clalit HMO, and having high perceived health literacy, efficacy and safety.

**Conclusion:**

Online and face-to-face healthcare services are complementary, while high-level and low-level OHS are distinct categories providing services for different healthcare needs. Because technological barriers are almost absent, access to OHS can expand more easily. Policymakers should focus on improving digital health literacy, particularly in the use of high-level technologies, and map the needs of the older population to provide them with personalized services.

## Background

Online health services (OHS) offer a potential solution to healthcare challenges arising from population aging, the increase in chronic diseases, and rising healthcare costs. They are perceived as a tool for improving the availability and accessibility of medical services and enhancing system efficiency (1–3). Additionally, they are considered a promising way to improve medical outcomes in various chronic conditions (4).

OHS have been found to provide an effective solution for secure provider–patient communication, with treatment outcomes comparable to, and sometimes even better than, those of face-to-face medical care. This is particularly evident in mental health assessment and treatment, rehabilitation counseling, and nutrition management (5). The definition of OHS includes the remote provision of healthcare services using information and communication technologies for diagnosis, treatment, and prevention, as well as for the continuous education and evaluation of healthcare providers (6). These tools encompass a wide range of applications, including two-way video conferencing, email, health apps, and other communication technologies.

The scope of medical treatment via OHS increased dramatically following the COVID-19 pandemic. However, despite their potential, these services are still characterized by technical and regulatory barriers that must be addressed to ensure broad and efficient implementation (7). These barriers include technical difficulties, resistance to change, costs, and limited financial reimbursement, as well as patient age and education level (2). These challenges are compounded by discomfort with technology adoption among both patients and healthcare providers, lack of OHS appeal, residence in urban areas (8), and ethical and regulatory complexities regarding data security and privacy (9). Dykgraaf et al. (7) found that, during the lockdowns of the COVID-19 period, despite the availability of video-based medical consultations, the public preferred using telephone consultations. According to their findings, this indicates that significant barriers to video-based OHS still exist. Therefore, it is necessary to address these perception-related issues to promote the desirable and feasible implementation of OHS among both healthcare providers and patients.

Patients with chronic illnesses have expressed high satisfaction with OHS. However, many still prefer face-to-face treatment despite their interest in using OHS (10, 11). Furthermore, OHS are perceived by patients as a complementary service to traditional medical care rather than a substitute (12). An Israeli study conducted during the COVID-19 pandemic found that most participants, including those with chronic conditions, preferred digital health services over visiting a clinic. They reported satisfaction with the service and expressed a positive intention to continue using digital health services in the future (11).

Health disparities linked are linked in Israel to sociodemographic characteristics such as age, sex, and nationality, as well as infrastructure gaps (in technological development and in other services) among regions and differences in healthcare service utilization among population groups (13). Evidence suggests that these disparities have widened in recent decades (14).

Despite one of the major advantages of OHS being improved accessibility to healthcare, differences in OHS usage exist among sectors. Minority groups and individuals from lower socioeconomic backgrounds tend to use these services less frequently. A study based on large databases from the COVID-19 period in the United States showed that, despite the availability of a wide range of OHS applications, disparities among population groups persisted (15). For example, adults aged 45–46 years were less likely to use these services than younger populations. Additionally, individuals living in urban areas were more likely to use OHS than those in rural regions. In England, research indicates that OHS have the potential to exacerbate health disparities between disadvantaged groups and the general population (14). While international research on OHS is growing, in Israel, there are few publications on the topic, particularly regarding their impact on specific populations (16).

A review of studies on OHS usage reveals a lack of information regarding its benefits, as well as a gap in understanding the impact of sociodemographic factors on OHS adoption and the elements that could encourage greater use of this technology (12). Further research is needed to better understand how variables such as age, sector, and influence the use of OHS.

## Methods

### Aims

(1) Describe the consumption of OHS and its characteristics by investigating knowledge, attitudes, barriers to use, consumption intentions, and usage patterns among various groups in Israel; (2) examine the relationships among knowledge, attitudes, barriers, and intentions to consume OHS; and (3) present a model for predicting OHS consumption based on the research variables.

### Study design

The present cross-sectional study included 2,001 Jewish and Arab participants aged 21 years and older. Sampling was conducted using stratified layers based on sectors and seven geographical districts, according to the classification of the Israel Central Bureau of Statistics, ensuring full nationwide coverage (North, Haifa, Tel Aviv, Central, Jerusalem, South, and Judea and Samaria). The sample represents the target population based on the variables of sex, sector, age group, and level of religiosity. Sampling within the different sectors and districts was conducted randomly.

All sampling quotas were pre-determined according to the proportions provided by the Central Bureau of Statistics and prior to data collection. iPanel implemented these sampling quotas during participant recruitment to ensure national representativeness.

### Study setting

Data collection was conducted online in September 2022 using the internet panel of iPanel, which consists of approximately 100,000 panel members.^1^ The research instrument was translated into Arabic by a certified translator following the academic principles of translation and back-translation. Participants were given the option to choose the questionnaire language.

Prior to data collection, a pilot study was conducted with 70 participants—40 from the Jewish sector and 30 from the Arab sector. Based on the pilot findings, the response scale for the barrier questionnaire was revised from two categories to three.

### Description of materials

The questionnaire included four parts.

#### Part A—attitudes, knowledge, and patient experience regarding the use of OHS

The final version of the questionnaire included 14 statements, rated on a 5-point Likert scale (1 = strongly disagree, 5 = strongly agree). Participants were asked to indicate their level of agreement with each statement. The validity and reliability of the instrument were established through an exploratory factor analysis (EFA). Following data collection, three factors were identified: effectiveness and confidence in online health services (OHS), online health literacy, and preference for face-to-face treatment (see Supplementary Table 1).

#### Part B—OHS barriers

This section included seven statements, each assessed independently. Because each statement stands alone, reliability analysis was not conducted.

- Technological barriers—Assessed through four statements, examining continuous and reliable internet access, availability of a laptop/desktop computer, and a smartphone.
- Emotional barriers—Assessed through three statements, examining the availability of services in the participant’s native language, confidence in using digital technologies, and willingness to engage in online communication with an unfamiliar healthcare provider.

#### Section C—familiarity with and frequency of OHS usage

This section consisted of 10 statements assessing familiarity with various OHS and the frequency of their use. Familiarity with the service was measured dichotomously (Yes/No). If the participant was familiar with the service, they were asked to report their frequency of use on a 1–6 scale: 1 = Never, 6 = Once a week or more.

Exploratory factor analysis identified two factors (see Supplementary Table 1):

1. Low-level technology (LLT)—Characterized by commonly accepted and widely used basic technologies.
2. High-level technology (HLT)—Characterized by more advanced technologies that require digital literacy.

#### Part D—sociodemographic background

This section included 15 items, covering demographic characteristics and personal details such as sex, age, sector, marital status, place of residence, sector, level of religiosity, and HMO affiliation. These variables were selected to ensure national representativeness and based on their well-established theoretical and empirical relevance as predictors of online health service (OHS) use and digital health technology consumption.

### Statistical analysis

Statistical analysis Data were analyzed using IBM SPSS Statistics version 29. First, the psychometric properties of the research instrument were assessed using exploratory factor analysis and Cronbach’s alpha coefficients for internal consistency. For each participant, an overall mean score was calculated for all questionnaire items, along with separate mean scores for each subscale, based on the relevant items within each questionnaire (where applicable). Mean scores were calculated for each subscale to create the study indices. Descriptive statistics, including means and standard deviations, were calculated to summarize the study variables and are presented as means and standard deviation. The normality of the data distribution was evaluated using skewness and kurtosis measures. Indices for all study variables ranged between −1 and +1 (Skewness: −0.40 to −0.15; Kurtosis: −0.44 to 0.30), indicating a normal distribution. Given the large sample size (*N* = 2,001), the use of parametric statistical tests was further supported by the Central Limit Theorem, which ensures that the sampling distribution of the mean approximates normality even when the underlying population distribution deviates modestly from normality.

Inferential statistics included Pearson correlation coefficients to examine relationships between variables, independent samples *t*-tests and one-way ANOVA to assess differences between groups (e.g., by sector, sex, and age), and hierarchical linear regression analyses were conducted to identify predictors of OHS familiarity and usage. Statistical significance was set at *p* < 0.05.

### Ethics approval

Ethical approval was obtained from the Ethics Committee of the Research Authority at Ono Academic College.

In Israel, the regulations for human subject research distinguish between interventional studies and studies based on existing data and questionnaires. This type of research is defined, among other things, as: “A prospective study in which health data is collected from individuals through direct contact with them.” Such studies do not require signed informed consent.^2^ Since this study is survey-based rather than interventional, the research questionnaire provided participants with a clear explanation of the study and the significance of their participation. Each participant had the option to decline participation or withdraw at any time. To safeguard participant rights, all participants were informed about the study details, including the estimated time required to complete the questionnaires. Furthermore, all collected data remained entirely anonymous and confidential. Completion of the questionnaire was considered as consent to participate.

## Results

### Participant characteristics

Table 1 describes the participants’ background characteristics. The mean was 47, ranging from 19 to 89 years (mean age, 47 years), and about half of the participants were women (50.20%). The majority were born in Israel, Jewish, secular, and affiliated with Clalit Health Services. Approximately half of the participants had an academic degree (48.42%), and most were employed (62.10%), married or in a relationship (69.5%), and resided in central Israel (27.94%).

**Table 1:** Sociodemographic characteristics of the study participants (*N* = 2,001).

Variable	Average	SD	
Age	47.07	16.81	
	Category	*N*	%
Age in categories	21–24	203	10.1
	25–34	423	21.1
	35–44	390	19.5
	45–54	352	17.6
	55–64	268	13.4
	65+	366	18.3
Sex	Men	996	49.80
	Women	1,005	50.20
Place of birth (*n* = 1742)	Israel	1,283	73.65
	Other	459	26.35
Health maintenance organization (HMO)	Clalit	1,022	51.10
	Maccabi	613	30.60
	Leumit	126	6.30
	Meuhedet	240	12.00
Education	Primary	12	0.06
	High school	1,017	51
	Academic	966	48.4
Sector	Jewish	1715	85.71
	Arabs	286	14.29
Level of religiosity (Jews only, *n* = 1711)	Secular	772	45.21
	Traditional	587	34.30
	Religious	176	10.20
	Ultra-orthodox	176	10.20

### Attitudes, knowledge, and patient experience regarding the use of OHS

Table 2 indicates that participants, on average, rated OHS as effective and safe (M =3.65, SD =0.65) and perceived themselves to have relatively high online health literacy (*M* = 3.79, SD = 0.73). However, the lowest score was recorded for comparison with face-to-face treatment (*M* = 3.35, SD = 0.64), with more than half of the respondents preferring in-person care over OHS.

**Table 2:** Familiarity with OHS.

	Query	Yes (%)
Low-level technology	Phone call with primary physician	94.5
	Scheduling an appointment via website or app	96.7
	Correspondence with primary physician via mail	63.9
	Phone call with a nurse	81.9
	Requesting prescriptions via website or app	90.3
High-level technology	Video call with primary physician	73.9
	Dietary consultation	33.8
	Telephone consultation with a specialist	62.5
	Online medical check-up	41.4
	Online mental health services	29.9

### Technological and emotional barriers

Approximately 90% of the participants reported no technological barriers, stating that they possess the necessary technological means to access OHS. Among the small percentage of respondents who lacked technological resources, most indicated that this did not prevent them from using OHS. A similar pattern was observed regarding emotional barriers. However, 11.7% of participants reported difficulty trusting a healthcare provider that they do not know and 10.4% stated that they do not want to consult an unfamiliar healthcare provider.

### Familiarity with and frequency of OHS use

Overall, a higher percentage of participants were familiar with LLT services (63.9–94.5%) compared with HLT services (26.1–70.1%). More than 50% of the participants were unfamiliar with HLT, which includes mental health consultations, nutrition counseling, and online medical examinations (Table 2). Additionally, when we analyzed familiarity by HMO affiliation, Clalit HMO members were found to be more familiar with HLT services such as *“Online medical examinations using* var*ious tools (*e.g*., blood pressure transmission, ECG, throat examination using the Tyto device)”* and less familiar with LLT services compared with members of other HMOs.

On average, the frequency of the use of LLT was approximately once every 3 months (*M* = 3.0, SD = 0.92). In contrast, the frequency of the use of HLT was once every 6 months or less frequently (*M* = 1.81, SD = 0.91). Additionally, more than 50% of participants familiar with HLT had never used these services, except for telephone consultations with a specialist, which were used by 68% of participants.

As can be seen in Table 3, several significant correlations were observed. For example, moderate to high positive correlations were found between effectiveness and confidence in OHS and Online health literacy (*r* = 0.67, *p* < 0.001). Positive low correlations were also found of effectiveness and confidence in OHS with familiarity with LLT (*r* = 0.22, *p* < 0.001) and Familiarity with HLT (*r* = 0.18, *p* < 0.001). Weak positive correlations were found of effectiveness and confidence in online treatment with familiarity with LLT (*r* = 0.22, *p* < 0.001) and familiarity with HLT (*r* = 0.18, *p* < 0.001). Weak to moderate positive correlations were also found of Online health literacy with familiarity with LLT (*r* = 0.29, *p* < 0.001), familiarity with HLT (*r* = 0.19, *p* < 0.001), and frequency of use of LLT (*r* = 0.18, *p* < 0.001). Weak negative correlations were found of technological barriers and emotional barriers with familiarity with LLT (*r* = −0.20 [*p* < 0.001] and *r* = −0.21 [*p* < 0.001], respectively). Moderate positive correlations were observed between familiarity with LLT and HLT (*r* = 0.39, *p* < 0.001) and frequency of use of LLT and HLT (*r* = 0.48, *p* < 0.001). Finally, a weak positive correlation was found between Preference for face-to-face treatment and emotional barriers (*r* = 0.19, *p* < 0.001).

**Table 3:** Pearson correlations among study variables.

	1	2	3	4	5	6	7	8	9
1	–								
2	0.67***	–							
3	−0.37***	−0.28***	–						
4	−0.17***	−0.22***	0.05*	–					
5	−0.30***	−0.31***	0.19***	0.27***	–				
6	0.22**	0.29***	−0.08***	−0.20***	0.21***	–			
7	0.18***	0.19***	−0.05*	−0.06**	−0.06	0.39***	–		
8	0.09***	0.18***	−0.01	0.02	0	0.02	0.07***	–	
9	0.04***	0.04	−0.04	0.11***	0.11***	−0.10***	0.07***	0.48***	–

*p < 0.05, **p < 0.01, ***p < 0.001. 1 = effectiveness and confidence in online treatment; 2 = online health literacy; 3 = preference for face-to-face treatment; 4 = technological barriers; 5 = emotional barriers; 6 = familiarity with LLT; 7 = familiarity with HLT; 8 = frequency of use of LLT; 9 = frequency of use of HLT.

### Differences among sociodemographic groups

Significant differences were found among groups in the study classified by sector, sex, and region of residence. The differences were analyzed using independent *t*-tests and one-way ANOVA.

In terms of sector, Jewish participants had higher perceived effectiveness and confidence in OHS (*M* = 3.70; SD = 0.62) than Arab participants (*M* = 3.41, SD = 0.67); *t*(370.41) = 6.84, *p* < 0.001.
Online health literacy was also higher among Jews (*M* = 3.85; SD = 0.71) than Arabs [*M* = 3.45, SD = 0.77; *t*(1,999) = 7.89, *p* < 0.001]. In addition, preference for face-to-face treatment was lower among Jews (*M* = 3.30; SD = 0.84) than Arabs [*M* = 3.69, SD = 0.76; *t*(409.27) = 7.90, *p* < 0.001]. Conversely, Arabs were more familiar with HLT (*M* = 2.66, SD = 1.69) than Jews [*M* = 2.37, SD = 1.48; *t*(362.69) = 2.71, *p* < 0.01] and reported more frequent use of HLT (*M* = 2.06, SD = 1.1) for Jews [*M* = 1.77, SD = 0.87; *t*(298.58) = 3.89, *p* < 0.001].
  Analysis by sex revealed that women reported higher online health literacy (*M* = 3.82, SD = 0.72) than men [*M* = 3.76, SD = 0.73; *t*(1,999) = 2.06, *p* < 0.05] and used LLT more often [*M* = 3.20, SD = 0.84 vs. 2.95, SD = 0.68; *t*(1,979) = 6.10, *p* < 0.001]. Similarly, men showed a stronger preference for face-to-face treatment (*M* = 3.40, SD = 0.84) than women [*M* = 3.31, SD = 0.83; *t*(1,999) = 2.30, *p* < 0.05].

Regarding region of residence, preference for face-to-face treatment was significantly higher among participants living in Northern Israel compared with other regions [*F*(6,1994) = 8.37, *p* < 0.001].

### Predicting familiarity and usage of HLT and LLT

Given the high levels of familiarity with and use of low-level technologies (LLT), predictive analyses focused on familiarity with and frequency of use of high-level technologies (HLT). Multiple linear regression models were applied to examine these outcomes. The models included effectiveness and confidence in online healthcare, online health literacy, preference for face-to-face treatment, age, familiarity with and frequency of LLT use, sector (Jewish/Arab), sex, and HMO affiliation as predictors. The regression results are presented in Tables 4, 5.

**Table 4:** Regression model predicting familiarity with HLT.

Variable	B	SE	t	Beta
Constant	200	277		
Efficacy and confidence in OHS	0.02	0.06	3.89***	0.10
Online health literacy	0.06	0.06	1.08	0.03
Preference for face-to-face care	−0.25	0.11	2.28*	−0.05
Age	−0.17	0.002	8.82***	−0.19
Familiarity with LLT	0.06	0.03	19.5***	0.41
Sector (Jewish = 1)	−0.46	0.09	5.04***	−0.10
Sex (Male = 1)	0.17	0.60	2.88**	0.06
Health fund—Leumit=1	−0.23	0.13	1.83	−0.04
Health fund—Meuhedet = 1	−0.21	0.01	2.14*	−0.04
Health fund—Maccabi = 1	−0.47	0.07	6.72***	−0.14

*R*^2^ = 0.23, *p* < 0.001, *f*(10, 1,990) = 59.72, *p* < 0.001. *p < 0.05, **p < 0.01, ***p < 0.001.

**Table 5:** Regression model predicting frequency of use of HLT.

Variable	*B*	SE	*t*	Beta
Constant	0.43	0.19		
Efficacy and confidence in OHS	0.10	0.04	2.48*	0.07
Online health literacy	−0.01	0.36	2.23*	−0.06
Preference for face-to-face care	0.03	0.02	1.31	0.03
Age	0.01	0.00	3.73***	−0.08
Frequency usage of LLT	0.49	0.02	23.47***	0.5
Sector (Jews = 1)	0.15	0.06	2.59**	−0.06
Sex (male = 1)	0.13	0.04	3.46***	0.07
Leumit health fund = 1	0.07	0.08	0.92	0.02
Meuhedet Health fund = 1	0.14	0.06	2.28*	−0.05
Maccabi health fund = 1	0.185	0.04	4.22***	−0.09

*R*^2^ = 0.261, *p* < 0.001, *f*(10, 1,760) = 62.02, *p* < 0.001. *p < 0.05, **p < 0.01, ***p < 0.001.

Prior to interpretation, all assumptions underlying multiple linear regression were examined and met. Nominal variables were coded using a K − 1 dummy variable approach, with the largest category serving as the reference group. Multicollinearity was ruled out, as variance inflation factor (VIF) values for all predictors were below 2.5. Independence of errors was confirmed using the Durbin–Watson statistic. Visual inspection of Normal P–P plots and scatterplots of standardized residuals against predicted values indicated that the assumptions of normality, linearity, and homoscedasticity were satisfactorily met.

From Table 4, it can be observed that the likelihood of familiarity with HLTs was increased among male users (*β* = 0.17, *p* < 0.01), Arabs (*β* = −0.46, *p* < 0.001), and those insured by Clalit Health Services. Additionally, the likelihood of familiarity with HLT increased with a greater sense of efficacy and confidence in online healthcare (*β* = 0.2, *p* < 0.001) and familiarity with LLT (*β* = 0.64, *p* < 0.001).

Conversely, the likelihood of familiarity with HLT decreased with increasing age (*β* = −0.17, *p* < 0.001), preference for face-to-face care (*β* = −0.025, *p* < 0.05), membership in Meuhedet Health Fund (*β* = −0.208, *p* < 0.05), and membership in Maccabi Health Fund (*β* = −0.473, *p* < 0.001). The predictive power of the model was 23%.

Table 5 shows that the frequency of HLT use was increased among male users (*β* = 0.07, *p* < 0.001), the Arab sector (*β* = −0.06, *p* < 0.05), and those insured by Clalit Health Services. Additionally, the frequency of HLT use increased with a higher perceived efficacy and confidence in online healthcare (*β* = 0.07, *p* < 0.05) and the frequency of LLT use (*β* = 0.5 *p* < 0.001).

Conversely, the frequency of HLT use decreased with lower online health literacy (*β* = −0.06, *p* < 0.05), increasing age (*β* = −0.08, *p* < 0.001), membership in Meuhedet Health Fund (*β* = −0.049, *p* < 0.05), and membership in Maccabi Health Services (*β* = −0.09, *p* < 0.001). The predictive power of the model was 26.1%.

## Discussion

The current study examined the factors influencing familiarity with and use of OHS in Israel, focusing on levels of digital health literacy, attitudes toward service quality, efficiency, and safety, and the relationship between sociodemographic characteristics and the frequency of use of these technologies. The primary contribution of this research lies in distinguishing between the use of LLTs (such as appointment scheduling and prescription retrieval) and HLTs (such as video consultations for diagnosis and counseling). It also deepens the understanding of the impact of emotional and technological barriers on the use of OHS. Additionally, the study identifies sectoral and age-related disparities and addresses minority groups that have not been adequately examined in previous research.

### Familiarity with and frequency of OHS usage

Online health technologies have numerous definitions, and research on this topic often refers to different technologies without specifying the exact ones involved. A unique contribution of the current study is its focus on two variables related to OHS consumption: the type of technology (high-level or low-level) and the familiarity with/frequency of the use of online health technologies. LLT refers to well-established technologies used for many years, mainly for administrative purposes, such as phone calls, emails, and prescription requests. In contrast, HLT involves video consultations, medical counseling, mental health services, diagnostics, and examinations.

The study found that, in general, a higher percentage of participants were familiar with OHS characterized as LLT compared to services categorized as HLT. Moreover, not everyone familiar with a technology necessarily used it. For example, approximately 70% of respondents were aware of video consultations with a physician, yet only about 45% reported using them at varying frequencies. A similar pattern was observed in mental health services, where familiarity and frequency of use were higher for LLT than for HLT.

On average, the frequency of LLT service use was once every 3 months, whereas the frequency of HLT service use was once every 6 months or more. Our results suggest that OHS usage frequency in Israel is higher than in other countries. A 2021 survey conducted in the United States found that only 37% of patients had used OHS at least once a year (17). Other studies report that, during the COVID-19 pandemic, the use of video-based telemedicine increased by 603%, particularly in emergency medicine and among younger patients (8, 18), although they do not provide detailed data on usage frequency.

Ebbert et al. (19) argue that prior experience with OHS increases the likelihood of future use. The findings of the current study indicate that HLT usage frequency is strongly linked to LLT usage frequency and is influenced by digital health literacy skills. Therefore, efforts to increase familiarity with and accessibility of OHS should focus on enhancing digital health literacy and raising awareness of HLT services. Encouraging the use of HLT services could facilitate a “leap forward” in diagnostics and treatment, making OHS more accessible to the general population.

### Technological and emotional barriers to OHS use

Most of the population has access to the technological means required to use OHS, and most are capable of utilizing the available services. This finding aligns with another study conducted during the COVID-19 pandemic in Israel, which reported that only 21% of respondents experienced technological difficulties with OHS (9). However, these findings contrast with the international literature, which highlighted barriers related to technical difficulties and resistance to change (2), as well as a lack of appeal and discomfort in using such services (8).

### Attitudes toward OHS

Attitudes toward OHS are influenced by three key factors: digital health literacy, perceived efficacy and confidence in online healthcare, and preference for face-to-face care. The present study found a strong positive correlation between perceived efficacy in online healthcare and digital health literacy, while both were negatively correlated with a preference for face-to-face care. This suggests that as confidence in OHS and digital health literacy increases, the public’s preference for in-person treatment decreases.

Digital health literacy and access to digital health technologies have been identified as key factors in determining the quality of healthcare services. In the current study, participants generally perceived themselves as having relatively high digital health literacy. However, about one-quarter of them only partially utilize online infrastructures for seeking information about illnesses and accessing health services, despite these services being available and accessible. Low health literacy, or an inability to understand medical terminology and patient instructions, may lead to poor adherence to treatment and medication misuse (20). Thus, the healthcare system faces the challenge of identifying populations in need of improved health literacy and focusing efforts on enhancing these skills.

### Perceived efficacy and confidence in OHS

OHS are perceived by the participants of the current study as being effective and varied, as they provide an additional treatment channel. This finding aligns with the increased adoption of digital healthcare services during and after the COVID-19 pandemic in Israel, where most participants preferred digital health services over in-person clinic visits (11). Similar trends have been reported in global studies (21, 22). From a policy perspective, it is crucial to continuously monitor OHS usage and public perceptions to further develop and expand these services as needed.

An examination of perceived efficacy and confidence in online healthcare reveals that public trust in some services remains relatively low. Only 30–40% of participants agreed that diagnosis and treatment via online technology yield results comparable to in-person consultations. Additionally, only about half of the respondents partially agreed that group therapy via computer makes treatment more accessible to those unable to attend in-person sessions. More than half of the respondents preferred face-to-face consultations with a physician or another healthcare provider (especially among Arab participants), and most had no strong opinion on the quality of OHS.

These findings are consistent with an Israeli study that found that both patients and physicians prefer in-person consultations over online video consultations (23). One possible explanation for this preference is the existing incentive model for physicians. A pre-COVID-19 study found that most patients rated OHS as either good or equivalent to in-person services, but only one-third preferred online consultations (22). The fact that perceptions of OHS efficacy, trust, and digital health literacy are relatively high in the current study, yet face-to-face treatment is still preferred, suggests that OHS should not replace in-person care but rather serve as a complementary service. This conclusion aligns with findings from similar studies (12).

In this context, researchers from the Mayo Clinic suggest that, given the advantages of digital health technologies, they should be integrated into traditional care to create virtual interactions between healthcare providers and patients between visits (24). Given these insights and the expansion of OHS policies, it is essential that health service providers, particularly HMOs, focus on strengthening public trust in these services and continuously monitoring their quality.

### Predictors of familiarity with and frequency of use of HLT

Given the finding that most participants are familiar with and use LLT, we conducted a multivariate regression analyses to identify the variables predicting familiarity and use of HLT exclusively. First, we found that familiarity with and frequency of use of HLT decreased with age. The literature presents conflicting findings regarding the relationship between age and digital technology use, with no clear distinction on the specific types of technology being used. Some studies have reported a negative correlation between age and online technology use (25), while another study found that adults older than 45 years were less likely to use digital health services (15). However, an Israeli study found that the use of LLT was similar in individuals aged 64–75 years and younger age groups in terms of usage frequency (26). These conflicting findings regarding OHS usage, particularly HLT, highlight the need for further research to understand the role of age in predicting engagement with these services.

Another predictor of HLT use and familiarity was Arab sector affiliation. This finding may seem unexpected, given that three-quarters of Arab respondents reported having low or significantly below-average incomes, residing mostly in peripheral areas, and being classified as a disadvantaged population. Studies in other countries have shown opposite findings, where populations living in urban areas were more likely to use OHS than those living in rural areas (14). Furthermore, ethnic minority populations from lower socioeconomic backgrounds have been found to use OHS less frequently (27).

A possible explanation for the findings regarding Arab participants in Israel is that their access to face-to-face healthcare services is more limited, compelling them to rely on HLT services. A relevant example is online dietitian consultations, which are classified as HLTs. Given the high rates of diabetes and obesity in the Arab population and the limited accessibility of dietitian services in peripheral areas, it is plausible that these constraints drive familiarity with and use of HLT services.

Men were found to have a higher likelihood than women of being familiar with and using HLTs, a finding consistent with other studies reporting gender differences in OHS consumption (15). Beyond gender-related demand-side differences, OHS could also help to reduce gender disparities in healthcare supply by enhancing women’s skills through support, supervision, proper guidance, and program development (28, 29).

Health fund membership was also found to be a significant predictor of OHS use. For example, membership in Clalit Health Services increased the likelihood of HLT familiarity and use. A possible explanation for this is that the survey included items on familiarity and use of diagnostic technologies associated with HLT, such as online medical check-ups using devices like blood pressure monitors, ECG transmission, and the Tyto device. Other health funds have primarily developed OHS focused on LLT. An Israeli study on HMO preferences for online physician consultations versus face-to-face care found that health funds expressed a need and desire to expand digital health services (23). These findings support the expansion of HLT services across all health funds.

Finally, perceived efficacy and confidence in online healthcare were also significant predictors of familiarity with and use of HLT. Additionally, the likelihood of HLT use decreased among those with lower digital health literacy. This underscores the critical role of digital health literacy in the adoption of HLTs. This finding is consistent with an Israeli study that identified digital health literacy as the strongest predictor of OHS usage (30).

### Policy implications and recommendations for decision-makers

- OHS should be categorized by service type

OHS include two distinct types: LLT and HLT. Therefore, OHS usage cannot be analyzed as a single entity. It is crucial to map these different service types across healthcare providers to better understand their implementation and accessibility.

- Focus on improving digital health literacy and HLT familiarity

Efforts to increase public familiarity with and accessibility of OHS should primarily focus on enhancing digital health literacy and expanding the use of HLTs. Greater adoption of HLT will enable significant advances in diagnostics and treatment, leveraging advanced monitoring devices with data transmission capabilities and virtual consultations, which are essential for managing various medical conditions.

- Leverage the existing technological infrastructure
- Given that the technological infrastructure is widely available in most households, the Ministry of Health, in collaboration with the “Digital Israel” initiative, can harness information and communication technologies to enhance the delivery of OHS (31).
- Integrate online and in-person healthcare services
- Online healthcare and face-to-face care are complementary services, not substitutes. Therefore, strategies should be developed to integrate the two approaches, tailoring them to different treatment processes, health conditions, and healthcare organizations.
- Age-specific adaptation of OHS
- Since familiarity with, and frequency of, OHS use decrease with age, OHS should not be the sole or primary care approach for older adults. Healthcare providers should assess the specific needs and capabilities of the older population and develop personalized service frameworks to ensure accessibility and usability.
- Further research on OHS use in the Arab sector. The findings regarding OHS use among the Arab population raise important questions and necessitate further research. While Arabs prefer face-to-face care and are less likely to use LLT, they demonstrate relatively high usage of HLT. Future studies should explore the underlying reasons for this pattern to better inform policy and service design.

### Study limitations

The limitations listed below should be acknowledged:

- Cross-sectional design—this design does not allow for causal inferences between the studied variables, but only for the identification of *statistical associations*. Future longitudinal studies could examine changes in online health service use over time and clarify the directionality of the relationships between attitudes, digital health literacy, and actual usage patterns.
- Self-reported data—the study relied on self-reported data collected through an online questionnaire, which may be subject to social desirability bias. Participants may have overestimated their digital health literacy or their familiarity with online health services, including high-level technologies (HLT), which could have influenced the precision of the reported usage patterns.
- Selection bias related to online recruitment—although the sample was nationally representative and stratified by key sociodemographic characteristics, the use of an internet-based panel may have resulted in the underrepresentation of individuals with limited internet access or very low digital skills, including very old adults and highly disadvantaged populations. Consequently, the low level of technological barriers reported in this study may underestimate barriers experienced by these groups.
- Limited assessment of clinical outcomes—the study focused on familiarity with and frequency of OHS use and did not include objective measures of service quality, clinical outcomes, or long-term patient satisfaction. Future studies integrating administrative data or clinical outcome measures could provide a more comprehensive evaluation of the effectiveness and impact of OHS.
- Evolving classification of online health services—the classification of online health services into low-level technologies (LLT) and high-level technologies (HLT), while a central contribution of this study, is based on a functional framework that may evolve as digital health services continue to develop. Ongoing validation and refinement of this classification will be important to maintain its relevance in future research.

## Conclusion

This study highlights unique aspects of the Israeli healthcare landscape and proposes practical solutions for expanding and tailoring the accessibility of OHS. It offers a novel perspective on OHS consumption in Israel, suggesting ways to effectively increase usage while addressing the specific needs of different population groups. While the study underscores the advantages of OHS, it also emphasizes the importance of integrating online and face-to-face services to enhance the efficiency and accessibility of the healthcare system.

OHS should not replace traditional care but rather complement it, necessitating the development of hybrid models that allow flexibility between online and in-person care based on patient needs and health conditions across different healthcare organizations.

By distinguishing between LLTs and HLTs, our results provide a new perspective on OHS familiarity and use in Israel. Policymakers and healthcare providers should not treat OHS as a single entity, but rather map the availability of these different service types across providers. Moreover, the lack of significant technological barriers identified in the study presents an opportunity for the Ministry of Health to further expand OHS. This should be done by enhancing digital health literacy and strengthening users’ confidence, particularly in adopting HLTs. Despite elevated access to digital technologies, some respondents reported difficulty consulting unfamiliar healthcare providers, indicating a need to enhance trust in OHS. Investment in tailored OHS solutions is especially critical for older adults, disadvantaged communities, and residents of peripheral areas. While younger populations tend to use HLT more frequently, older adults continue to prefer face-to-face care.

Finally, our findings regarding OHS usage among the Arab population highlight disparities that require further research to better understand their specific needs and barriers. Identifying these challenges will be essential in ensuring equitable access to digital healthcare services.

## Data Availability

The raw data supporting the conclusions of this article will be made available by the authors, without undue reservation.

## Footnotes

1
www.ipanel.co.il

2
https://www.gov.il/blobFolder/guide/protocol-of-medical-research-involving-human-subjects/he/clinical_trials_cth_GLOSSARY.pdf
