# Supplementary material for: The digital transformation of healthcare: a national cross-sectional study on usage, patterns, attitudes, and barriers across social groups in Israel
Source: Front Public Health. 2026 Jan 12;13:1735502. doi: 10.3389/fpubh.2025.1735502 (PMC12833286; doi:10.3389/fpubh.2025.1735502)
Supplement: Supplementary file 1 [file Table_1.docx]

Supplementary

Table A**. Exploratory factor analysis results and psychometric properties of the study instrument**

| **Factor / Variable** | **Cronbach's α** | **Items / Statements** | **Factor Loading** |
| --- | --- | --- | --- |
| Efficacy and confidence in OHS | 0.76 | 1. Medical diagnosis via online technology provides results that are just as accurate as those obtained in a face-to-face consultation. | 0.69 |
|  |  | 2. Group therapy via computer makes treatment accessible to patients unable to attend in-person sessions. | 0.65 |
|  |  | 3. Consuming healthcare services via digital technology saves time. | 0.65 |
|  |  | 4. Digital technology adds additional options for communicating with the care team in a reasonable time. | 0.63 |
|  |  | 5. Video communication with a doctor is a good solution for treating infants and children. | 0.51 |
|  |  | 6. I feel safe receiving treatment from a doctor via a phone or computer session. | 0.49 |
| Online health literacy | 0.8 | 1. I already use all the digital communication means my HMO has to offer. | 0.75 |
|  |  | 2. I know how to use online technology to define my health problems. | 0.73 |
|  |  | 3. I tend to search for information about diseases and treatments on websites. | 0.71 |
|  |  | 4. I intend to continue consuming digital medical services in the future. | 0.59 |
|  |  | 5. Digital medical services make it easier for me to receive medical treatment. | 0.54 |
| Preference for face-to-face treatment | 0.74 | 1. I prefer to receive face-to-face treatment with the caregiver at the clinic. | 0.88 |
|  |  | 2. I always prefer a direct (frontal) meeting with the doctor. | 0.87 |
|  |  | 3. Communicating with healthcare providers via online technology will reduce the quality of medical care. | 0.61 |
| High-level technology (HLT) | 0.93 | 1. Received mental health services (psychiatry, psychotherapy, social work) via a phone call. | 0.86 |
|  |  | 2. Performed an online medical check-up using various tools (e.g., blood pressure transmission, ECG, throat examination using Tyto device). | 0.85 |
|  |  | 3. Received nutritional and personal diet services via a phone call. | 0.84 |
|  |  | 4. Received telephone consultation from a specialist doctor. | 0.7 |
|  |  | 5. Conducted a video call with the family doctor. | 0.66 |
| Low-level technology (LLT) | 0.8 | 1. Scheduled an appointment via the HMO website or app. | 0.86 |
|  |  | 2. Requested prescriptions for various medications via the HMO website or app. | 0.83 |
|  |  | 3. Conducted a phone call with the family doctor. | 0.54 |
|  |  | 4. Corresponded with the family doctor via email. | 0.52 |
|  |  | 5. Conducted a phone call with a nurse. | 0.48 |
